# Wing morphometrics as a possible tool for the diagnosis of the *Ceratitis fasciventris*, *C. anonae*, *C. rosa* complex (Diptera, Tephritidae)

**DOI:** 10.3897/zookeys.540.9724

**Published:** 2015-11-26

**Authors:** Joannes Van Cann, Massimiliano Virgilio, Kurt Jordaens, Marc De Meyer

**Affiliations:** 1 University of Antwerp (Evolutionary Ecology Group), Groenenborgerlaan 171, B-2020 Antwerp, Belgium; 2 University of Jyväskylä, PO Box 35, 40014 Finland; 3 Royal Museum for Central Africa (Invertebrates Unit & JEMU), Leuvensesteenweg 13, B-3080 Tervuren, Belgium

**Keywords:** *Ceratitis anonae*, *Ceratitis fasciventris*, *Ceratitis rosa*, fruit flies, cryptic species, integrative taxonomy, wing morphometrics, Posterior Group Membership Probability

## Abstract

Previous attempts to resolve the *Ceratitis* FAR complex (*Ceratitis fasciventris*, *Ceratitis anonae*, *Ceratitis rosa*, Diptera, Tephritidae) showed contrasting results and revealed the occurrence of five microsatellite genotypic clusters (A, F1, F2, R1, R2). In this paper we explore the potential of wing morphometrics for the diagnosis of FAR morphospecies and genotypic clusters. We considered a set of 227 specimens previously morphologically identified and genotyped at 16 microsatellite loci. Seventeen wing landmarks and 6 wing band areas were used for morphometric analyses. Permutational multivariate analysis of variance detected significant differences both across morphospecies and genotypic clusters (for both males and females). Unconstrained and constrained ordinations did not properly resolve groups corresponding to morphospecies or genotypic clusters. However, posterior group membership probabilities (PGMPs) of the Discriminant Analysis of Principal Components (DAPC) allowed the consistent identification of a relevant proportion of specimens (but with performances differing across morphospecies and genotypic clusters). This study suggests that wing morphometrics and PGMPs might represent a possible tool for the diagnosis of species within the FAR complex. Here, we propose a tentative diagnostic method and provide a first reference library of morphometric measures that might be used for the identification of additional and unidentified FAR specimens.

## Introduction

“True” fruit flies (Diptera, Tephritidae) are represented by more than 4,000 phytophagous species of which 25–30% feed on fruits. Many of them include major agricultural pests affecting crop production (Aluja and Norrbom 1999). Fruit fly pests also include a number of economically important species complexes that cannot be adequately resolved by morphological or molecular characters (Schutze et al. 2012, Schutze et al. 2015a, Selivon et al. 2005, Virgilio et al. 2008, Virgilio et al. 2013) and for which pest management proves to be problematic (e.g. see Schutze et al. 2015a). These include the so-called *Ceratitis* FAR complex, a small group of morphologically similar species including *Ceratitis fasciventris* (Bezzi, 1920), *Ceratitis anonae* Graham, 1908 and the main agricultural pest *Ceratitis rosa* Karsch, 1887 (the Natal fruit fly). Males of these species can be morphologically separated using leg ornamentation patterns (De Meyer and Freidberg 2006) while females of *Ceratitis rosa* and *Ceratitis fasciventris* cannot be morphologically resolved (De Meyer 2001b). The diagnosis of the three species is further complicated by their partially overlapping geographic distributions (Copeland et al. 2006, De Meyer 2001a) and by the fact that *Ceratitis fasciventris* and *Ceratitis rosa* might not even comply to the biological species concept as they produce fertile hybrids under laboratory conditions (Erbout et al. 2008).

Previous attempts to separate the three morphospecies using alternative diagnostic methods showed contrasting results. Both PCR - RFLP (Barr et al. 2006) and sequencing of mitochondrial and nuclear DNA markers (Virgilio et al. 2008) could not resolve the three morphospecies as separate entities. Analyses of cuticular hydrocarbon profiles (Vaníčková et al. 2014), however, showed differences between colony samples of *Ceratitis fasciventris*, *Ceratitis anonae* and *Ceratitis rosa* (a single population for each species) and suggested that this might also reflect differences between morphospecies. Microsatellites (Virgilio et al. 2013) failed to separate the three species as individual entities but showed the existence of five genotypic clusters largely corresponding to the morphospecies *Ceratitis anonae* (cluster A), *Ceratitis fasciventris* (clusters F1 and F2) and *Ceratitis rosa* (clusters R1 and R2). Interestingly, *a posteriori* morphological analyses of *Ceratitis rosa* types R1 and R2 (Virgilio et al. 2013) revealed differences in male secondary sexual characters (shape of, and extent of coloration on, midtibia). Additionally, the two *Ceratitis rosa* types showed significantly different distributions along an altitudinal transect in Tanzania (Mwatawala et al. 2015) thus suggesting possible ecological differentiation between genotypic clusters.

This paper aims at exploring morphometric differentiation between FAR morphospecies and genotypic clusters in an integrative taxonomic framework (see Schutze et al. 2015b and references therein). In particular, we used wing morphometrics to verify the diagnosis of a group of *Ceratitis* FAR specimens that were characterised previously by morphology and microsatellite genotyping.

## Methods

### Study material

We considered a set of 227 specimens genotyped at 16 polymorphic microsatellites (Virgilio et al. 2013). The set included 80 *Ceratitis anonae*, 97 *Ceratitis fasciventris* and 50 *Ceratitis rosa* individuals of both sexes assigned to five genotypic clusters (see Suppl. material 1 and 2 for specimen details). Only specimens with at least one intact wing were used. Wings were mounted on a glass slide (dorsal side facing up) and a 2mm ladder was used for scaling. Digital images were taken using a Micropublisher 5.0 RTV camera (QImaging, Canada) mounted on a Leica MZ12.5 microscope. Two pictures (10x magnification) were taken with the first picture focusing on the wing and the second on the scale placed over the glass slide. The two pictures were then merged using the program Auto-Montage (Syncroscopy, Cambridge, UK). Seventeen homologous type I landmarks (Bookstein 1997) consisting of points at which a wing vein meets the edge of the wing, or wing vein and cross-vein intersections were selected. Thirteen of the landmarks were homologous to those of Schutze et al. (2011), and an additional four were chosen to try to obtain a higher resolution of species and of genotypic clusters. No landmarks were chosen in the anterior and posterior regions of the wing because these areas are most easily damaged. Six partial wing band areas were also selected and digitalised (Suppl. material 3, 4). All wing band areas were entirely within a single wing cell or were separated by wing veins and cross-veins. Some wing band areas did not border a vein (e.g. anterior part of area 3), and thus should be considered as semi-landmarks according to Gunz et al. (2005). ImageJ (Abramoff et al. 2004) was used to score wing landmark coordinates and measure wing band areas. Wing landmark coordinates were scaled, translated, and rotated against the consensus configuration in MorphoJ (Klingenberg 2011) according to the generalized least squares Procrustes superimposition method (Rohlf 1999).

### Statistical analyses

A preliminary methodological experiment aimed at quantifying morphometric differences between sexes (see Gidaszewski et al. 2009), left and right wings as well as of possible biases related to measurement errors (Arnqvist and Martensson 1998). For this purpose, a subset of 7 male and 7 female *Ceratitis rosa* specimens were randomly selected and morphometric measures (both of wing landmarks and wing band areas) were replicated by taking two digital images of each left and right wing and by scoring each digital image twice. Permutational Multivariate Analysis of Variance (PERMANOVA, Anderson 2001, 2005) was used to test the effects of (a) sex (as fixed factor), (b) wing (fixed orthogonal factor), (c) digital image (random factor nested in wing) and (d) measurement (random factor nested in the interaction of wing x image). As this test confirmed the occurrence of morphometric differences between sexes (see Results), we decided to separately consider males (163 specimens) and females (64 specimens see discussion). Whenever possible, right wings were used for digital imaging (this regardless of the lack of correlation between wing side and morphometrics, see results) however, right wings were damaged in 19.3% of specimens and in those cases left wings were used. Principal Component Analysis (PCA), as implemented by the R package *adegenet* 1.4-2 (Jombart 2008), was used to visualise differences in male and female wing landmarks and wing band areas across morphospecies (*Ceratitis fasciventris*, *Ceratitis anonae* and *Ceratitis rosa*) and genotypic clusters (F1, F2, R1, R2 A). Prior to PCA, data were centred by the *scaleGen* function of *adegenet*. Morphospecies and genotypic clusters were then considered as prior groups and specimens ordinated by maximising between-group variances through Discriminant Analysis of Principal Components (DAPC). The number of Principal Components (PCs) retained in DAPCs was optimised through the *xvalDapc* function of *adegenet* (Jombart et al. 2010). *XvalDapc* was then used to calculate average individual posterior group membership probabilities (PGMPs) to morphospecies and genotypic clusters (for both wing landmarks and wing band areas). *XvalDapc* performs replicated DAPCs on training sets including 90% of specimens (selected through stratified random sampling) and calculates PGMPs of the remaining 10%. For these analyses, 1000 replicates were carried out at each level of PC retention. The proportion of consistently assigned specimens, i.e. of specimens for which the highest PGMP corresponded to the prior specimen grouping (with respect to morphospecies and genotypic clusters) were quantified by considering (a) the highest PGMP of each specimen (no probability threshold) and (b) two arbitrary thresholds corresponding to PGMP = 0.95 and PGMP = 0.99. In this latter case, only specimens with PGMPs higher than the thresholds were assigned, the other ones were discarded. PERMANOVAs (Anderson 2001) were performed on male and female specimens (for wing landmarks and wing band areas) and independently tested differences across (a) morphospecies (fixed factor) and (b) genotypic cluster (fixed factor). In order to avoid biases associated to the unbalanced experimental design (see Anderson and Walsh 2013), PERMANOVAs were performed on random subsets of data including balanced numbers of replicates (ranging from 3 to 31 replicate specimens). Tests were based on 10^4^ unrestricted permutations of Euclidean distances between untransformed Procrustes coordinates. *A posteriori* pair-wise comparisons on significant terms were also implemented using the PERMANOVA t-statistic (Anderson 2005). Probability values of repeated pair-wise tests were corrected for Type I errors using the False Discovery Rate (FDR) procedure (Benjamini and Hochberg 1995).

## Results

### Preliminary methodological control

PERMANOVAs revealed differences in multivariate patterns of 7 male and 7 female *Ceratitis rosa* specimens for both wing landmarks and wing band areas (Tab. 1) while it neither detected significant effects related to wing (left or right) nor to measurement error (both for repeated images of the same wing or for repeated scoring of the same image). Similarly, PCAs of wing landmarks of the *Ceratitis rosa* specimens used in the methodological test (Suppl. material 5) showed separation between males and females, while measures of (a) left and right wings, (b) repeated images and (c) repeated scoring were largely overlapping (see 95% confidence ellipses). PCAs of wing band areas (Suppl. material 6) did not allow separating male from female control specimens. A PCA of wing landmarks of all 227 specimens also suggested sex related differences across morphospecies (see further and Suppl. material 7).

**Table 1.** Preliminary methodological control. PERMANOVA testing differences in multivariate patterns of wing landmarks and wing band areas of 14 *Ceratitis rosa* specimens in response to: sex (male / female), wing (right / left), image (1, 2) and measure (A, B). d.f.: degrees of freedom; MS: mean square estimates; F: pseudo-F. Probability of Monte Carlo simulations: n.s.: not significant a P<0.05; ***: P<0.001.

		wing landmarks			wing band areas		
	d.f.	MS	F		MS	F	
Sex = S	1	0.0220	68.64	***	0.1390	66.23	***
Wing = W	1	0.0001	0.30	n.s.	0.0006	0.28	n.s.
Image = I(W)	2	0.0000	0.10	n.s.	0.0011	0.52	n.s.
Measure = M(IxW)	4	0.0000	0.03	n.s.	0.0001	0.05	n.s.
S x W	1	0.0001	0.45	n.s.	0.0006	0.29	n.s.
S x I(W)	2	0.0000	0.06	n.s.	0.0001	0.03	n.s.
S x M(WxI)	4	0.0000	0.04	n.s.	0.0001	0.04	n.s.
Residual	96	0.0003			0.0021		

### Morphometrics of morphospecies and genotypic clusters

The first two PC axes of PCAs including all specimens accounted for 57.9% and 47.6% of variation in males and females, respectively. PCAs of both wing landmarks (Suppl. material 8) and wing band areas (Suppl. material 9) did not resolve either morphospecies or genotypic clusters. DAPC analyses were based on 10 and 20 PCs (for males and females, respectively) when considering wing landmarks and morphospecies as a prior group, on 10 (for males) and 5 (for females) PCs when considering wing landmarks and genotypic cluster and prior group and on 6 PCs (for both males and females) when using wing band areas and morphospecies or genotypic cluster as prior variables. Stressing the ordination of points by using prior groups did not provide a better resolution of both morphospecies and genotypic clusters and DAPC 95% confidence ellipses were largely overlapping (Suppl. material 10, Suppl. material 11).

**Figure 1.**
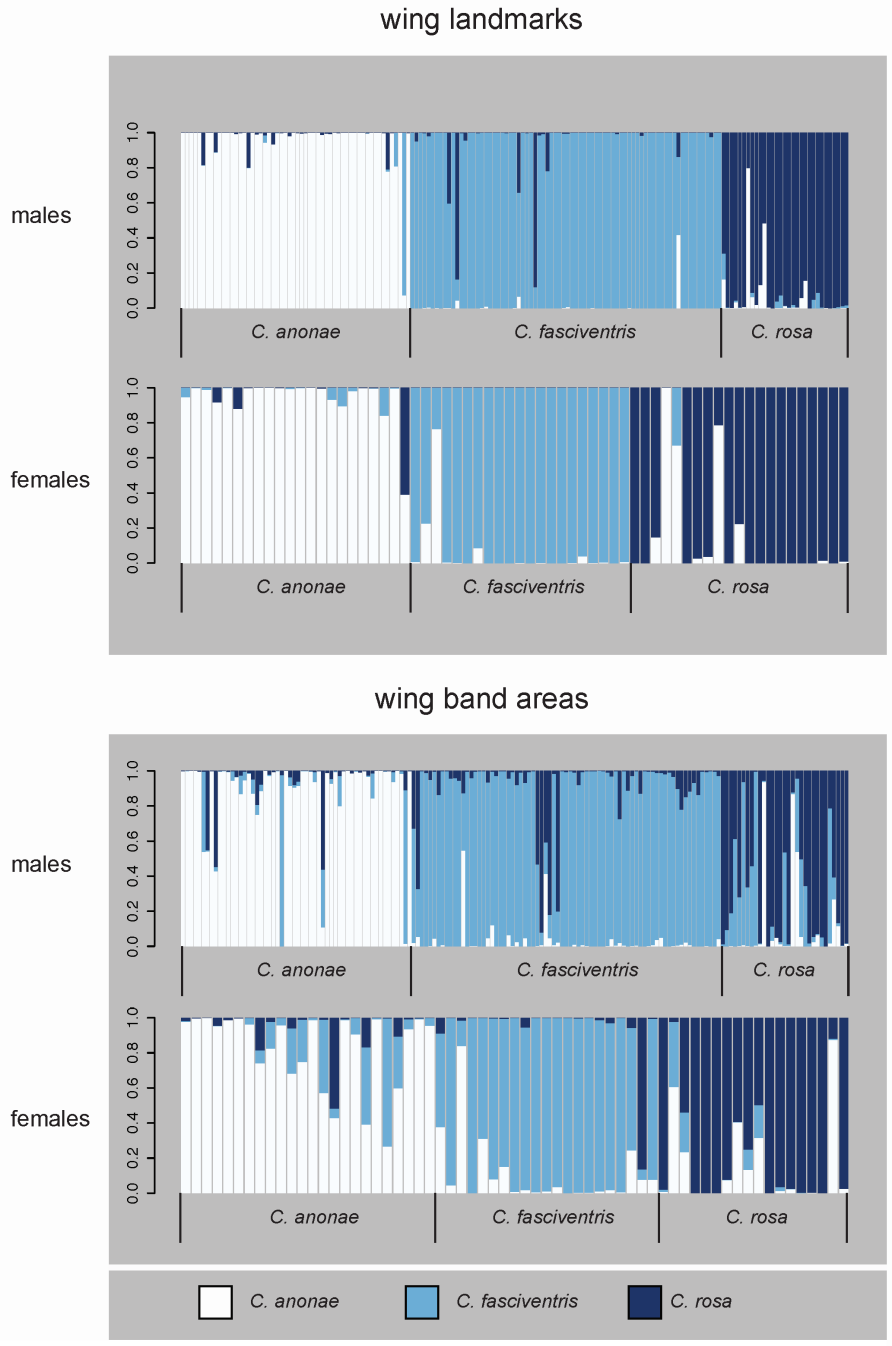
Individual assignments to morphospecies. Posterior group membership probabilities (PGMPs) of male and female specimens as resulting from Discriminant Analysis of Principal Coordinates of wing landmarks (upper) or wing band areas (lower). Prior groups: *Ceratitis anonae* (white), *Ceratitis fasciventris* (light blue), *Ceratitis rosa* (dark blue).

**Figure 2.**
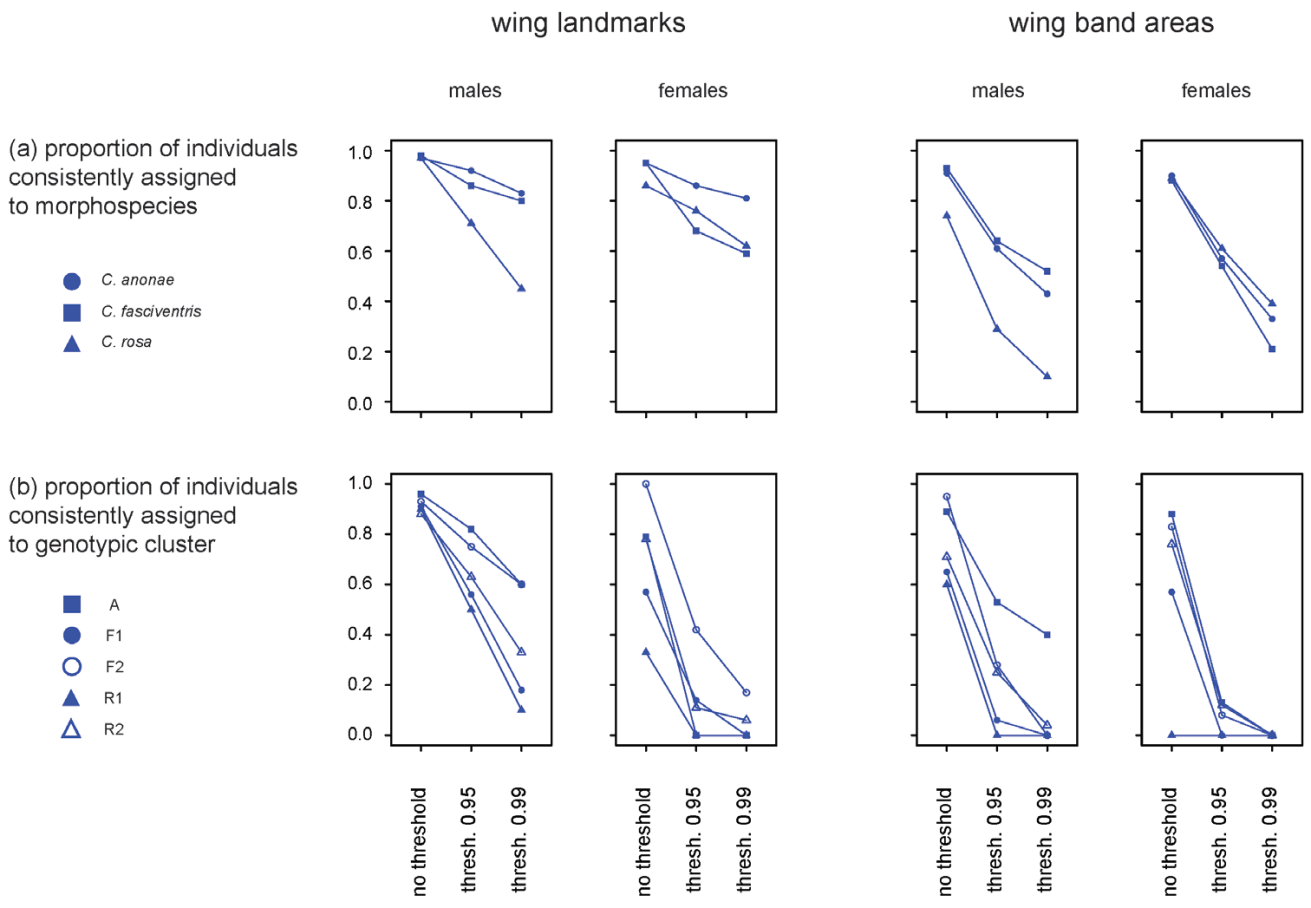
Diagnostic performance at different identification thresholds. Proportions of male and female specimens consistently assigned (**a**) to morphospecies (i.e. of specimens for which the highest posterior group membership probabilities (PGMP) corresponds to the prior morphospecies grouping) and (**b**) to genotypic cluster (i.e. of specimens for which the highest PGMP corresponds to the prior A, F1, F2, R1, R2 genotypic grouping) when considering wing landmarks (left) or wing band areas (right) and by using different assignment thresholds (no threshold, PGMP = 0.95, PGMP = 0.99).

The average individual PGMP within morphospecies (Figure 1, Suppl. material 12) ranged from 0.85 (SD = 0.33, females *Ceratitis rosa*) to 0.95 (SD = 0.13, males *Ceratitis anonae*) for wing landmarks and from 0.68 (SD = 0.35, males *Ceratitis rosa*) to 0.89 (SD = 0.24, males *Ceratitis anonae*) for wing band areas. Average PGMP of genotypic groups (Suppl. material 12, Suppl. material 13) was more variable and ranged from 0.27 (SD = 0.2, females R1) to 0.94 (SD = 0.19, males A) for wing landmarks and from 0.11 (SD = 0.04, females R1) to 0.86 (SD = 0.26, males A). When using wing landmarks, the proportion of specimens consistently assigned to morphospecies (Figure 2) ranged from 0.45 (*Ceratitis rosa* males, probability threshold = 0.99) to 0.98 (*Ceratitis anonae* males, no threshold), when considering wing band areas from 0.10 (*Ceratitis rosa* males, threshold = 0.99) to 0.93 (*Ceratitis anonae* males, no threshold). The proportion specimens consistently assigned to genotypic cluster (Figure 2) was highly variable and ranged from 0.0 (females A, threshold 0.95, females F1 and R1, threshold 0.99) to 1.0 (females F2, no threshold) when considering wing landmarks and from 0.0 (females R1, no threshold, females F1, threshold 0.95, males F1, F2, R1, females A, F2, R2, threshold 0.99) to 94.9 (males F2, no threshold) when considering wing band areas.

PERMANOVAs evidenced significant interspecific differences in both wing landmarks and wing band areas (for both males and females, Table 2, Suppl. material 14). *A posteriori* comparisons showed significant pairwise differences across all species in male and female wing band areas and in female wing landmarks. Conversely, male wing landmarks differed only between *Ceratitis anonae* and *Ceratitis rosa*. PERMANOVA also showed differences across genotypic clusters (but with different patterns in *a posteriori* pairwise comparisons within males and females and between wing landmarks and wing band areas (Table 3, Suppl. material 15).

**Table 2.** Morphometric differences across morphospecies (wing landmarks).

PERMANOVA and *a posteriori* comparisons (t-statistic) testing differences in multivariate patterns of wing landmarks among morphospecies (*Ceratitis anonae*, *Ceratitis fasciventris*, *Ceratitis rosa*). d.f.: degrees of freedom; MS: mean square estimates; F: pseudo-F. Probability of Monte Carlo simulations: n.s.: not significant a P<0.05; ***: P<0.001, **: P<0.01; *: P<0.05 (after False Discovery Rate Correction for repeated *a posteriori* comparisons).								
	**males**				**females**			
	**d.f.**	**MS**	**F**		**d.f.**	**MS**	**F**	
Morphospecies	2	0.0021	2.34	*	2	0.0039	6.63	***
Residual	90	0.0009			54	0.0006		
								

Pair-wise *a posteriori* comparisons (males and females: lower and upper diagonal matrix, respectively).			
	*Ceratitis anonae*	*Ceratitis fasciventris*	*Ceratitis rosa*
*Ceratitis anonae*	-	***	*
*Ceratitis fasciventris*	n.s.	-	***
*Ceratitis rosa*	*	n.s.	-

**Table 3.** Morphometric differences across genotypic clusters (wing landmarks).

PERMANOVA and *a posteriori* comparisons (t-statistic) testing differences in multivariate patterns of wing landmarks among genotypic clusters (A, F1, F2, R1, R2). d.f.: degrees of freedom; MS: mean square estimates; F: pseudo-F. Probability of Monte Carlo simulations: n.s.: not significant a P<0.05; ***: P<0.001, **: P<0.01; *: P<0.05 (after False Discovery Rate Correction for repeated *a posteriori* comparisons).								
	**males**				**females**			
	d.f.	MS	F		d.f.	MS	F	
Genotypic clusters	4	0.0037	6.10	***	4	0.0013	2.30	**
Residual	45	0.0006			10	0.0006		

Pair-wise *a posteriori* comparisons (males and females: lower and upper diagonal matrix, respectively)					
	A	F1	F2	R1	R2
A	-	n.s.	n.s.	n.s.	n.s.
F1	***	-	*	n.s.	n.s.
F2	***	n.s.	-	*	n.s.
R1	n.s.	**	**	-	n.s.
R2	**	***	**	**	-

## Discussion

The preliminary methodological experiment showed significant differences between male and female *Ceratitis rosa* wing morphometrics and suggests that these differences are consistent across species. Wing size and shape is supposed to have a main role in visual and vibrational courtship displays of tephritid fruit flies and sexual dimorphism in wing morphometrics has been already shown in tephritids (e.g. Marsteller et al. 2009). Once established the occurrence of relevant sexual variation (in at least one of the target species), we separately considered males and females. This approach allowed (a) disentangling sexual differences in wing shape from morphospecies / genotypic related variation and (b) maximising the number of balanced male and female replicates used in PERMANOVA.

PERMANOVAs showed that *Ceratitis fasciventris*, *Ceratitis anonae*, *Ceratitis rosa* as well as their five genotypic clusters (Virgilio et al. 2013), differ in their wing shape and banding patterns. These differences did not seem particularly pronounced, as (un)constrained ordinations neither resolved morphospecies nor genotypic clusters. However, PGMPs of wing landmarks (and particularly of male wing landmarks) proved to be effective in consistently assigning a large proportion of individuals to morphospecies. Interestingly, compared to microsatellite genotyping (Virgilio et al. 2013), wing landmarks seem more efficient in resolving the three FAR morphospecies as separate entities. Wing landmarks allowed a consistent species identification of a relevant proportion of males (97–98%, depending on morphospecies) even if the percentage of consistently identified females was lower (86–95%). The performance of wing band areas for species identification is poorer, particularly for male *Ceratitis rosa* (where only 74% of specimens could be consistently identified). The disagreement between morphological, morphometric and genotypic data was further evidenced by the tentative diagnosis of the genotypic clusters which showed large variations in the proportion of consistently assigned specimens and much poorer identification performances.

Distance thresholds are currently implemented in DNA barcoding identification of species (Ratnasingham and Hebert 2013, Sonet et al. 2013) where they can heavily affect identification performances (Virgilio et al. 2012). Similarly to what previously proposed for DNA barcoding, implementing PGMP thresholds, might help reducing the proportion of false positives (i.e. of individuals erroneously assigned to a morphospecies / genotypic cluster) as it would only allow the morphometric identification of specimens with reasonably high membership probabilities (e.g. > 0.95 or > 0.99), while for all other ones, alternative / complementary identification methods might be considered. Considering PGMP thresholds in the morphometric identification of specimens also caused a marked reduction of the proportion of consistently identified specimens (due to individuals that were discarded from identification as not reaching the threshold). It would now be interesting to verify if relationships between PGMP thresholds and identification success are similar to what has already been observed between distance thresholds and DNA barcoding identification performance, where more restrictive identification thresholds could increase identification precision but negatively affect accuracy (Virgilio et al. 2012).

## Conclusion

This study suggests that wing morphometrics might represent a possible tool for the diagnosis of species within the *Ceratitis* FAR complex. In this respect, PGMP of individuals might be calculated and used to quantify the proximity of individuals to each morphospecies (see Jombart et al. 2010). The morphometric measures collected for the 227 FAR specimens considered here (and provided as supplementary material) might be used as a reference library to calculate group memberships of additional and unidentified FAR specimens. A larger reference dataset, including more representatives of each morphospecies and genotypic cluster, is now required to properly quantify relationships between PGMP thresholds and identification accuracy and precision.

## Supplementary materials

Supplementary material 1 Morphometric data Data type: morphometric data Explanation note: Specimen list and details and raw morphometric data (for both wing landmarks and wing band areas). This dataset is made available under the Open Database License (http://opendatacommons.org/licenses/odbl/1.0/). The Open Database License (ODbL) is a license agreement intended to allow users to freely share, modify, and use this Dataset while maintaining this same freedom for others, provided that the original source and author(s) are credited.Joannes Van Cann, Massimiliano Virgilio, Kurt Jordaens, Marc De Meyer

Supplementary material 2 Map of sampling locations Data type: occurence Explanation note: Number of sampled specimens for each morphospecies are indicated in parentheses. This dataset is made available under the Open Database License (http://opendatacommons.org/licenses/odbl/1.0/). The Open Database License (ODbL) is a license agreement intended to allow users to freely share, modify, and use this Dataset while maintaining this same freedom for others, provided that the original source and author(s) are credited.Joannes Van Cann, Massimiliano Virgilio, Kurt Jordaens, Marc De Meyer

Supplementary material 3 Wing landmarks and wing band areas Data type: species data Explanation note: List of wing landmarks and wing band areas considered in this study. This dataset is made available under the Open Database License (http://opendatacommons.org/licenses/odbl/1.0/). The Open Database License (ODbL) is a license agreement intended to allow users to freely share, modify, and use this Dataset while maintaining this same freedom for others, provided that the original source and author(s) are credited.Joannes Van Cann, Massimiliano Virgilio, Kurt Jordaens, Marc De Meyer

Supplementary material 4 Wing landmarks and wing band areas Data type: species data Explanation note: Position of wing landmarks and wing band areas (numbers according to Suppl. material 3). This dataset is made available under the Open Database License (http://opendatacommons.org/licenses/odbl/1.0/). The Open Database License (ODbL) is a license agreement intended to allow users to freely share, modify, and use this Dataset while maintaining this same freedom for others, provided that the original source and author(s) are credited.Joannes Van Cann, Massimiliano Virgilio, Kurt Jordaens, Marc De Meyer

Supplementary material 5 Preliminary methodological experiment: unconstrained ordination of wing landmarks Data type: species data Explanation note: Principal component analysis (PCA) showing morphometric differences in wing landmarks of 14 Ceratitis rosa specimens across sexes (M, F), wings (LW: left wing, RW: right wing), repeated images of the same wing (1, 2), repeated measures of the same image (A, B). This dataset is made available under the Open Database License (http://opendatacommons.org/licenses/odbl/1.0/). The Open Database License (ODbL) is a license agreement intended to allow users to freely share, modify, and use this Dataset while maintaining this same freedom for others, provided that the original source and author(s) are credited.Joannes Van Cann, Massimiliano Virgilio, Kurt Jordaens, Marc De Meyer

Supplementary material 6 Preliminary methodological experiment: unconstrained ordination of wing band areas Data type: species data Explanation note: Principal component analysis (PCA) showing morphometric differences in wing band areas of 14 Ceratitis rosa specimens across sexes, wings (LW: left wing, RW: right wing), repeated images of the same wing (1, 2), repeated measures of the same image (A, B). This dataset is made available under the Open Database License (http://opendatacommons.org/licenses/odbl/1.0/). The Open Database License (ODbL) is a license agreement intended to allow users to freely share, modify, and use this Dataset while maintaining this same freedom for others, provided that the original source and author(s) are credited.Joannes Van Cann, Massimiliano Virgilio, Kurt Jordaens, Marc De Meyer

Supplementary material 7 Unconstrained ordination of wing landmarks across sexes of each morphospecies Data type: species data Explanation note: Principal component analysis (PCA) showing morphometric differences in wing landmarks between sexes of each morphospecies (*Ceratitis anonae*, *Ceratitis fasciventris*, *Ceratitis rosa*). (all 227 specimens included). This dataset is made available under the Open Database License (http://opendatacommons.org/licenses/odbl/1.0/). The Open Database License (ODbL) is a license agreement intended to allow users to freely share, modify, and use this Dataset while maintaining this same freedom for others, provided that the original source and author(s) are credited.Joannes Van Cann, Massimiliano Virgilio, Kurt Jordaens, Marc De Meyer

Supplementary material 8 Unconstrained ordination of wing landmarks Data type: species data Explanation note: Principal component analysis (PCA) showing morphometric differences in wing landmarks between males and females (a) *Ceratitis anonae*, *Ceratitis fasciventris* and *Ceratitis rosa* and (b) genotypic clusters A, F1, F2, R1, R2. This dataset is made available under the Open Database License (http://opendatacommons.org/licenses/odbl/1.0/). The Open Database License (ODbL) is a license agreement intended to allow users to freely share, modify, and use this Dataset while maintaining this same freedom for others, provided that the original source and author(s) are credited.Joannes Van Cann, Massimiliano Virgilio, Kurt Jordaens, Marc De Meyer

Supplementary material 9 Unconstrained ordination of wing band areas Data type: species data Explanation note: Principal component analysis (PCA) showing morphometric differences in wing band areas between males and females (a) *Ceratitis anonae*, *Ceratitis fasciventris* and *Ceratitis rosa* and (b) genotypic clusters A, F1, F2, R1, R2. This dataset is made available under the Open Database License (http://opendatacommons.org/licenses/odbl/1.0/). The Open Database License (ODbL) is a license agreement intended to allow users to freely share, modify, and use this Dataset while maintaining this same freedom for others, provided that the original source and author(s) are credited.Joannes Van Cann, Massimiliano Virgilio, Kurt Jordaens, Marc De Meyer

Supplementary material 10 Constrained ordination of wing landmarks Data type: species data Explanation note: Discriminant analysis of principal coordinates (DAPC) maximising morphometric differences in wing landmarks between males and females (a) *Ceratitis anonae*, *Ceratitis fasciventris* and *Ceratitis rosa* and (b) genotypic clusters A, F1, F2, R1, R2. This dataset is made available under the Open Database License (http://opendatacommons.org/licenses/odbl/1.0/). The Open Database License (ODbL) is a license agreement intended to allow users to freely share, modify, and use this Dataset while maintaining this same freedom for others, provided that the original source and author(s) are credited.Joannes Van Cann, Massimiliano Virgilio, Kurt Jordaens, Marc De Meyer

Supplementary material 11 Constrained ordination of wing band areas Data type: species data Explanation note: Discriminant analysis of principal coordinates (DAPC) maximising morphometric differences in wing band areas between males and females (a) *Ceratitis anonae*, *Ceratitis fasciventris* and *Ceratitis rosa* and (b) genotypic clusters A, F1, F2, R1, R2. This dataset is made available under the Open Database License (http://opendatacommons.org/licenses/odbl/1.0/). The Open Database License (ODbL) is a license agreement intended to allow users to freely share, modify, and use this Dataset while maintaining this same freedom for others, provided that the original source and author(s) are credited.Joannes Van Cann, Massimiliano Virgilio, Kurt Jordaens, Marc De Meyer

Supplementary material 12 Average individual assignments Data type: species data Explanation note: Average individual posterior group membership probabilities (PGMPs) of male and female specimens of three morphospecies (*Ceratitis anonae*, *Ceratitis fasciventris* and *Ceratitis rosa*) and five genotypic clusters (A, F1, F2, R1, R2) as resulting from the analysis of wing landmarks (light grey) and wing band areas (dark grey). This dataset is made available under the Open Database License (http://opendatacommons.org/licenses/odbl/1.0/). The Open Database License (ODbL) is a license agreement intended to allow users to freely share, modify, and use this Dataset while maintaining this same freedom for others, provided that the original source and author(s) are credited.Joannes Van Cann, Massimiliano Virgilio, Kurt Jordaens, Marc De Meyer

Supplementary material 13 Individual assignments to genotypic clusters A, F1, F2, R1, R2 Data type: molecular data Explanation note: Posterior group membership probabilities (PGMPs) of male and female specimens as resulting from Discriminant Analysis of Principal Coordinates of wing landmarks (upper) or wing band areas (lower). Prior groups: A, F1, F2, R1, R2 (from white to dark blue). This dataset is made available under the Open Database License (http://opendatacommons.org/licenses/odbl/1.0/). The Open Database License (ODbL) is a license agreement intended to allow users to freely share, modify, and use this Dataset while maintaining this same freedom for others, provided that the original source and author(s) are credited.Joannes Van Cann, Massimiliano Virgilio, Kurt Jordaens, Marc De Meyer

Supplementary material 14 Morphometric differences across morphospecies (wing band areas) Data type: morphometric data Explanation note: PERMANOVA and a posteriori comparisons (t-statistic) testing differences in multivariate patterns of wing band areas among morphospecies (*Ceratitis anonae*, *Ceratitis fasciventris*, *Ceratitis rosa*). d.f.: degrees of freedom; MS: mean square estimates; F: pseudo-F. Probability of Monte Carlo simulations: n.s.: not significant a P<0.05; ***: P<0.001, **: P<0.01; *: P<0.05 (after False Discovery Rate Correction for repeated a posteriori comparisons). This dataset is made available under the Open Database License (http://opendatacommons.org/licenses/odbl/1.0/). The Open Database License (ODbL) is a license agreement intended to allow users to freely share, modify, and use this Dataset while maintaining this same freedom for others, provided that the original source and author(s) are credited.Joannes Van Cann, Massimiliano Virgilio, Kurt Jordaens, Marc De Meyer

Supplementary material 15 Morphometric differences across genotypic clusters (wing band areas) Data type: morphometric data Explanation note: PERMANOVA and a posteriori comparisons (t-statistic) testing differences in multivariate patterns of wing band areas among morphospecies (*Ceratitis anonae*, *Ceratitis fasciventris*, *Ceratitis rosa*). d.f.: degrees of freedom; MS: mean square estimates; F: pseudo-F. Probability of Monte Carlo simulations: n.s.: not significant a P<0.05; ***: P<0.001, **: P<0.01; *: P<0.05 (after False Discovery Rate Correction for repeated a posteriori comparisons). This dataset is made available under the Open Database License (http://opendatacommons.org/licenses/odbl/1.0/). The Open Database License (ODbL) is a license agreement intended to allow users to freely share, modify, and use this Dataset while maintaining this same freedom for others, provided that the original source and author(s) are credited.Joannes Van Cann, Massimiliano Virgilio, Kurt Jordaens, Marc De Meyer
