# Supplementary figures and images for: Wing morphometrics as a possible tool for the diagnosis of the Ceratitis fasciventris, C. anonae, C. rosa complex (Diptera, Tephritidae)
Source: Zookeys. 2015 Nov 26;(540):489–506. doi: 10.3897/zookeys.540.9724 (PMC4714084; doi:10.3897/zookeys.540.9724)

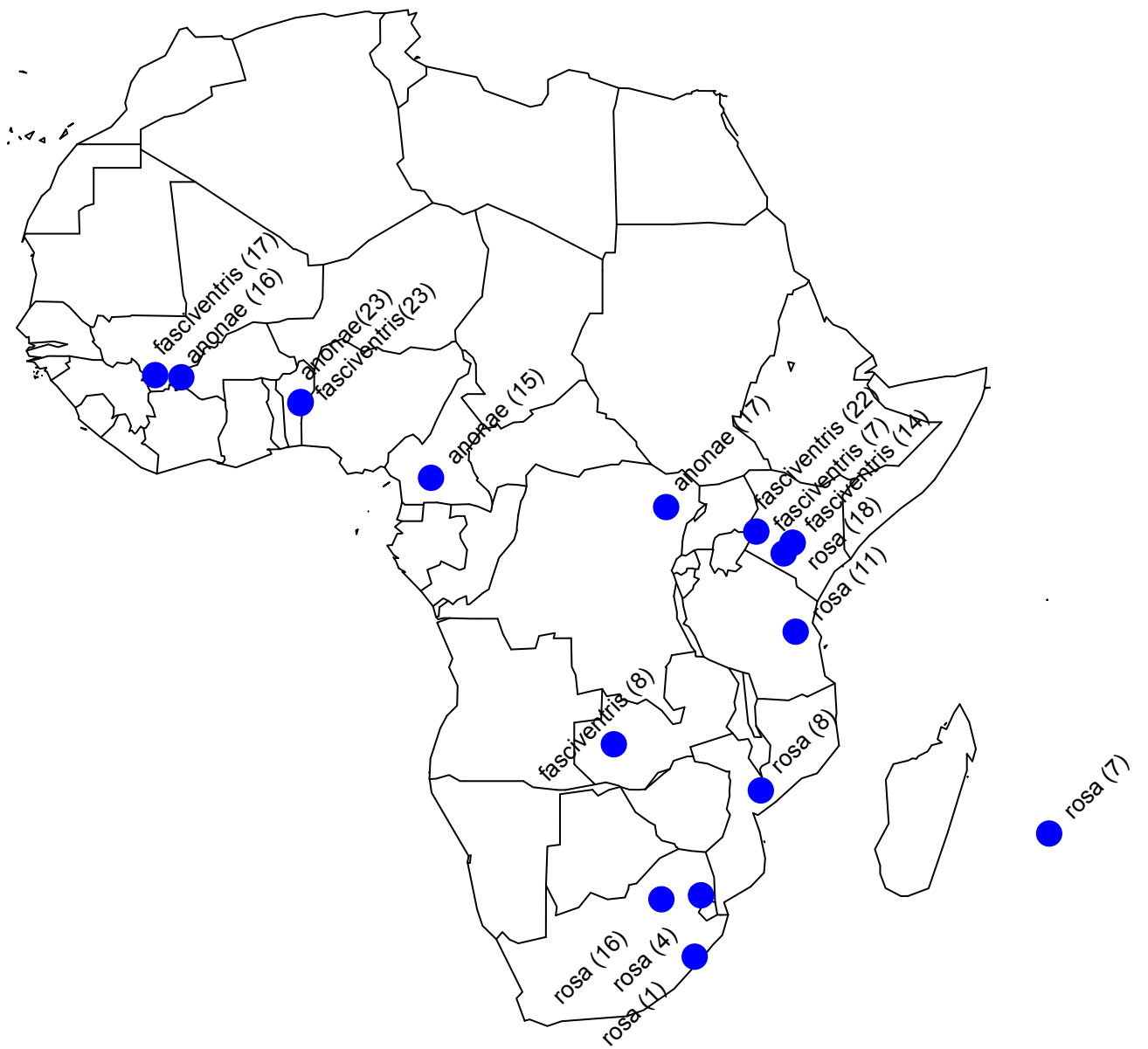

Supplement: Supplementary material 2 — Map of sampling locations [file zookeys-540-489-s002.pdf]

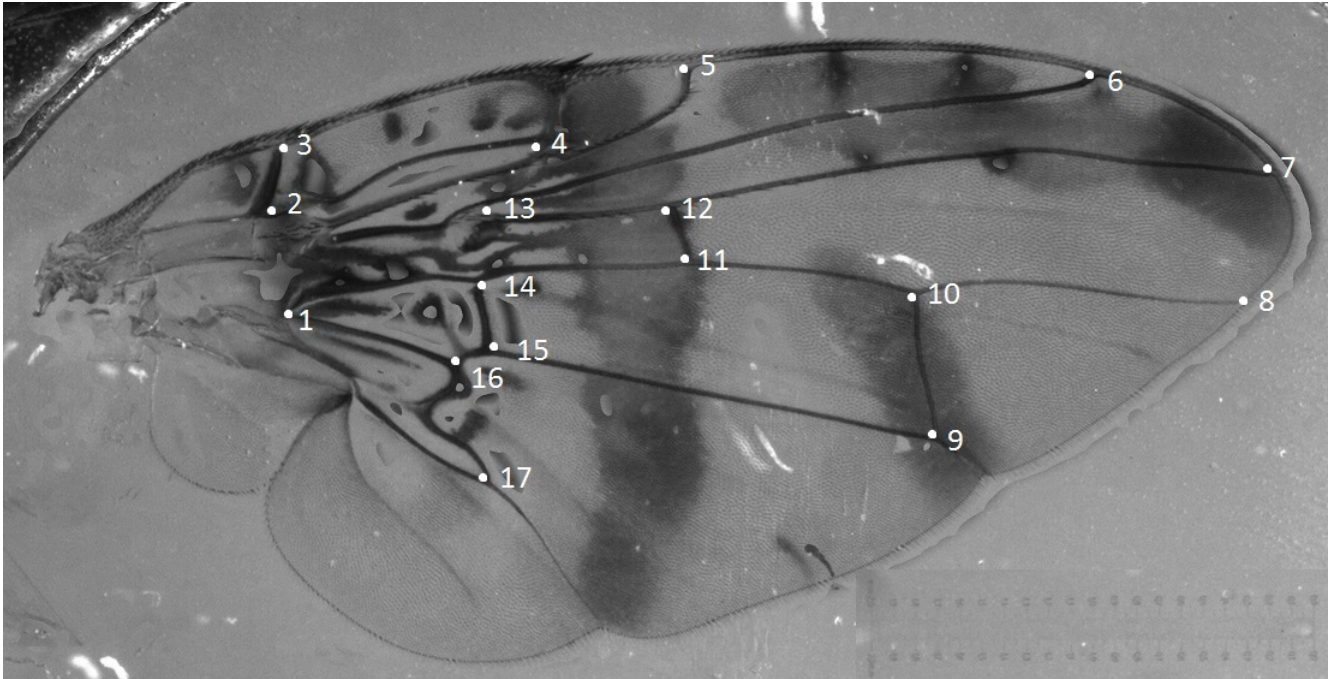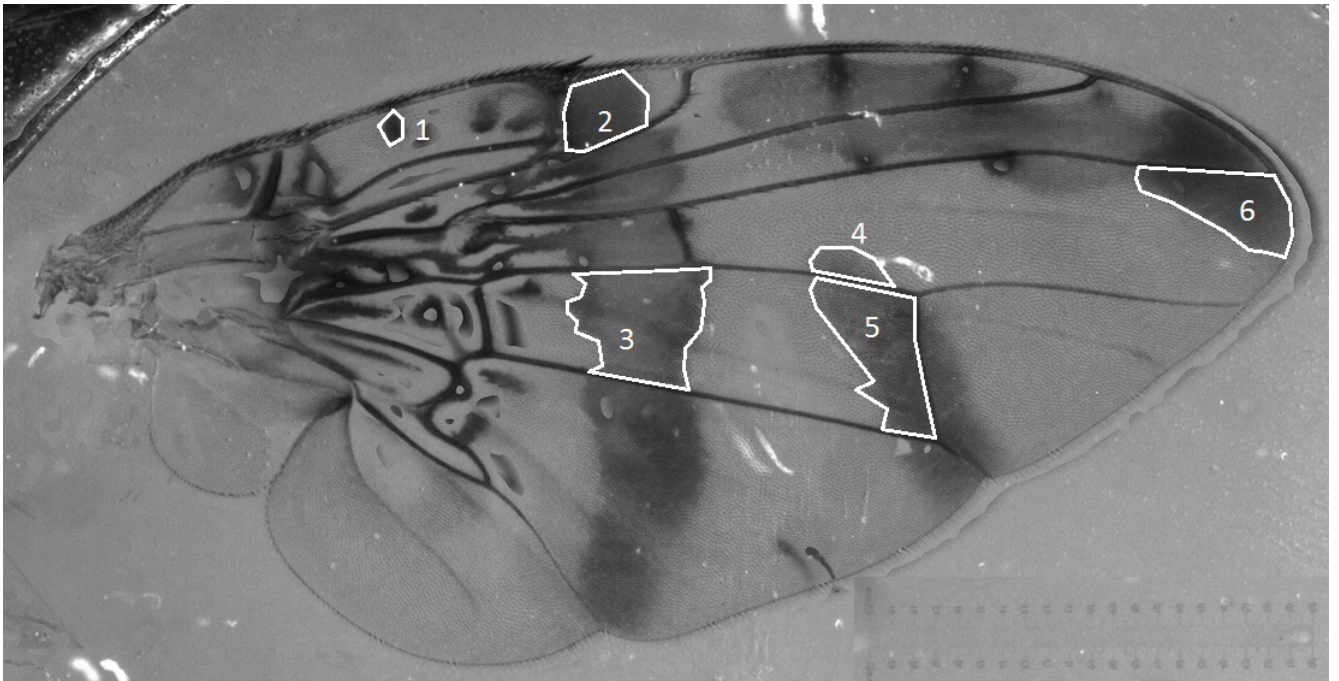

Supplement: Supplementary material 4 — Wing landmarks and wing band areas [file zookeys-540-489-s004.pdf]

methodological control:  
wing landmarks *C. rosa*

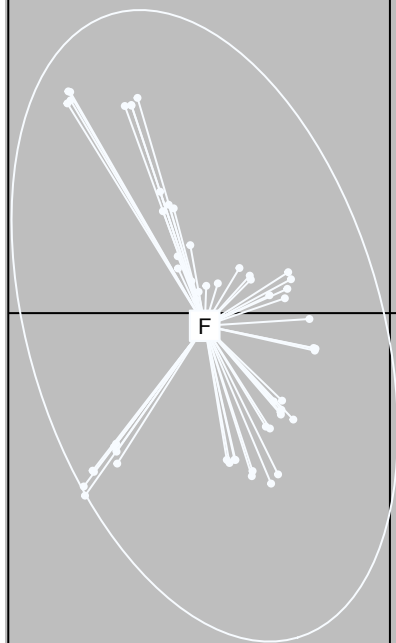

sex

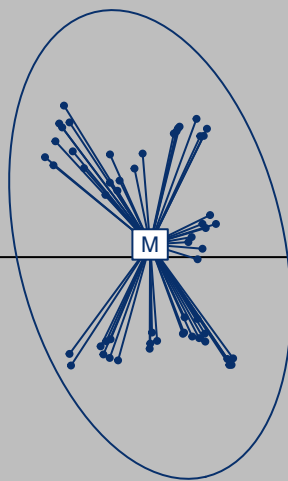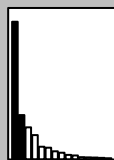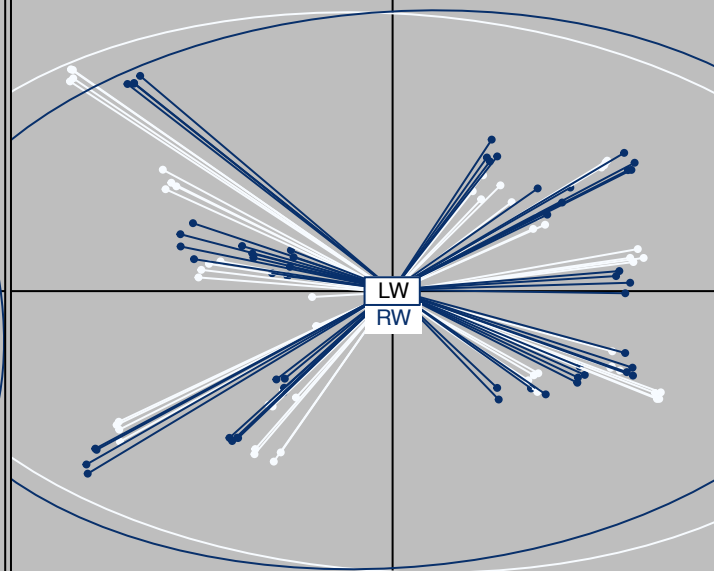

wing

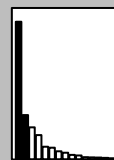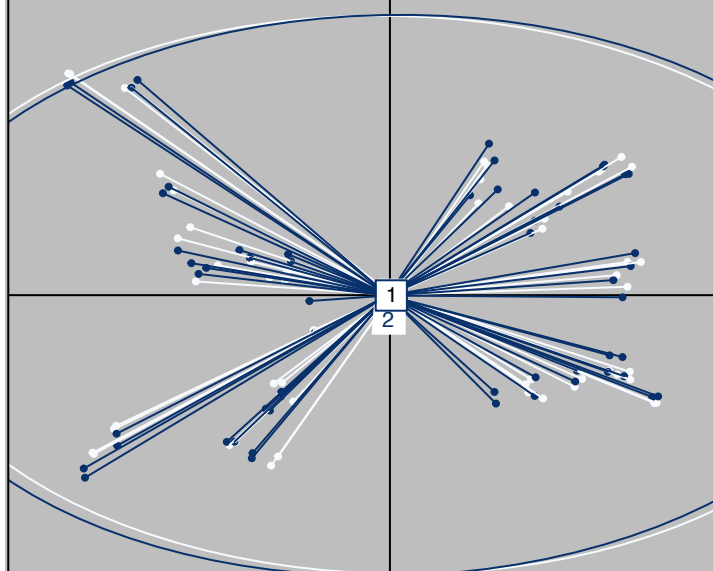

image

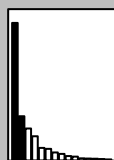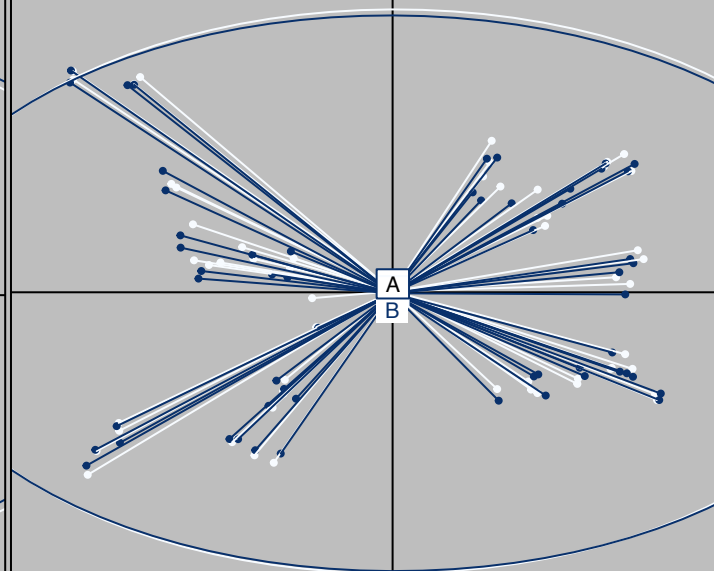

measure

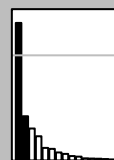

Supplement: Supplementary material 5 — Preliminary methodological experiment: unconstrained ordination of wing landmarks [file zookeys-540-489-s005.pdf]

*methodological control:*  
*wing band areas C. rosa*

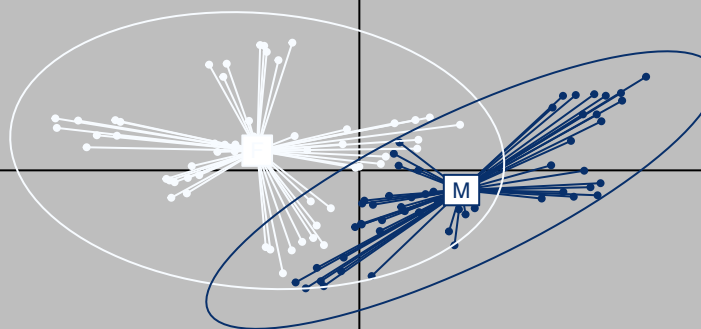

sex

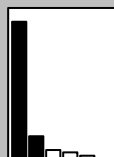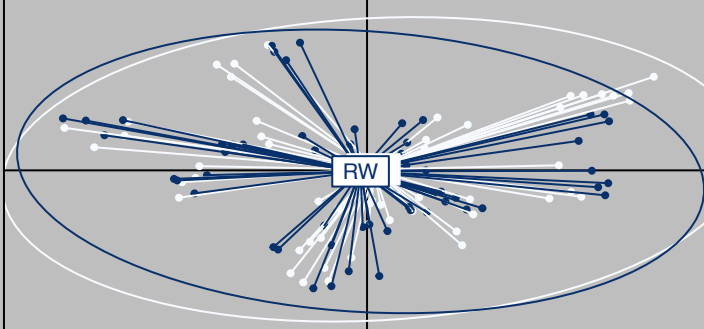

wing

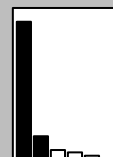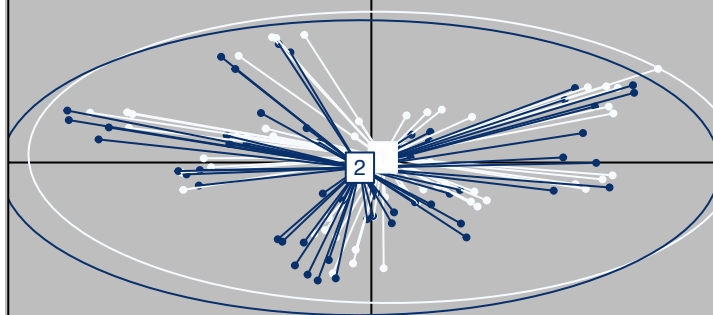

image

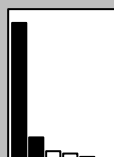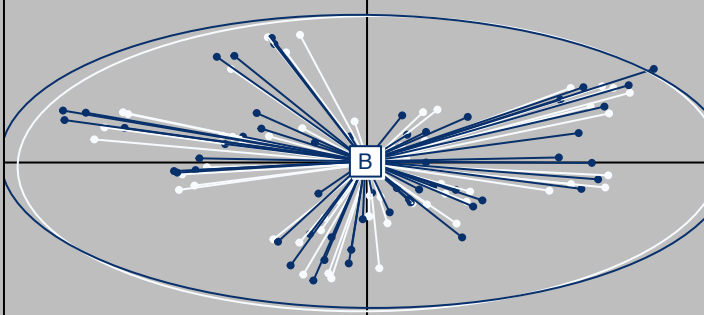

measure

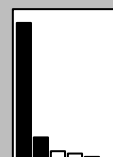

Supplement: Supplementary material 6 — Preliminary methodological experiment: unconstrained ordination of wing band areas [file zookeys-540-489-s006.pdf]

# wing landmarks (all specimens)

*C. anonae*

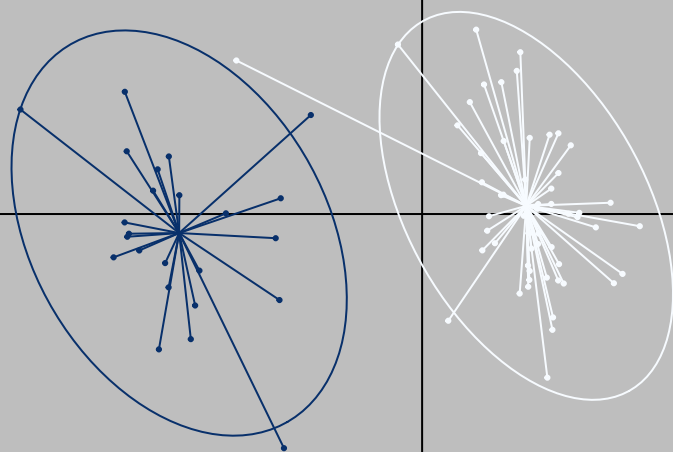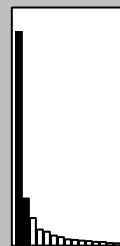

*C. rosa*

□ Males  
■ Females

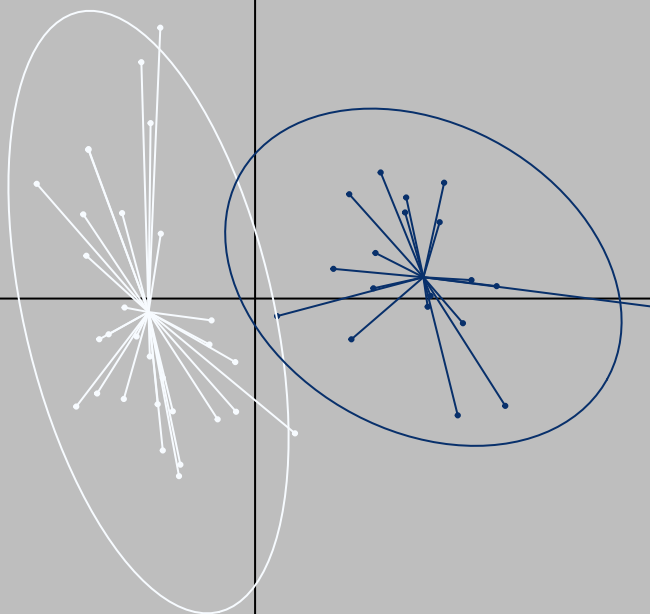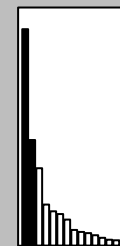

*C. fasciventris*

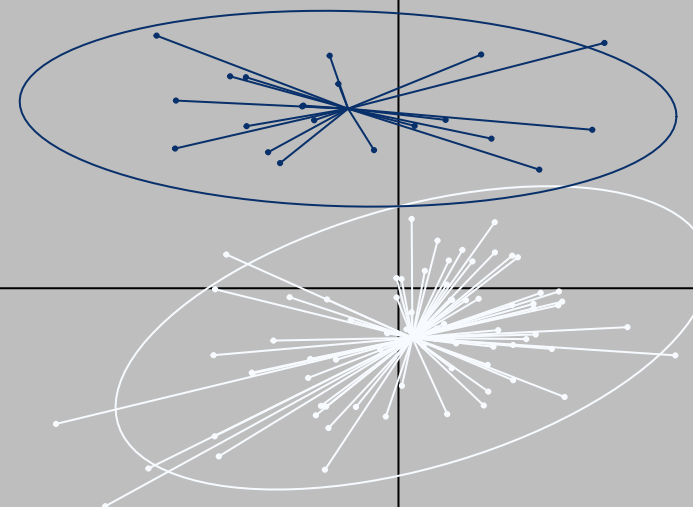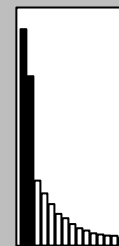

Supplement: Supplementary material 7 — Unconstrained ordination of wing landmarks across sexes of each morphospecies [file zookeys-540-489-s007.pdf]

PCA wing band areas

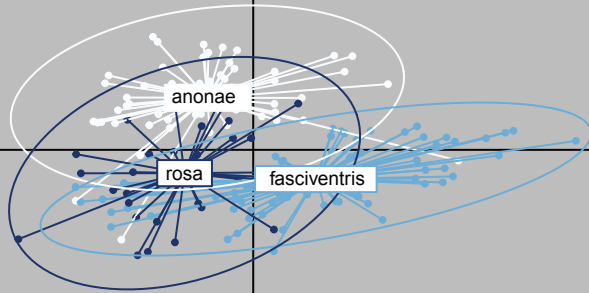

males

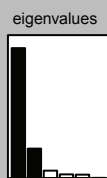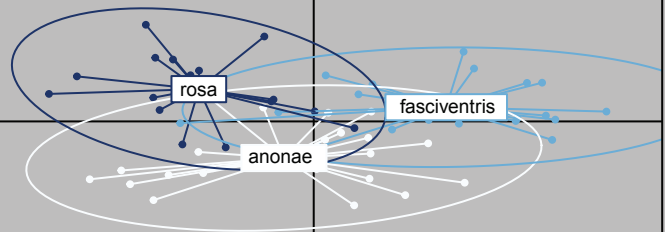

females

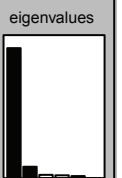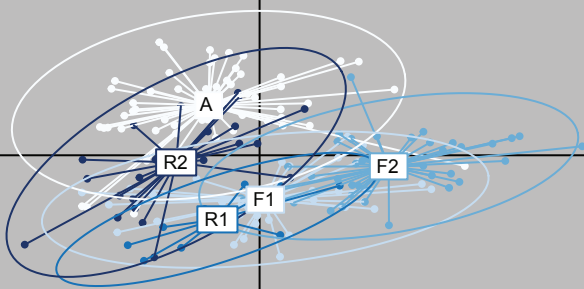

males

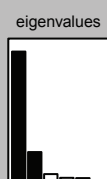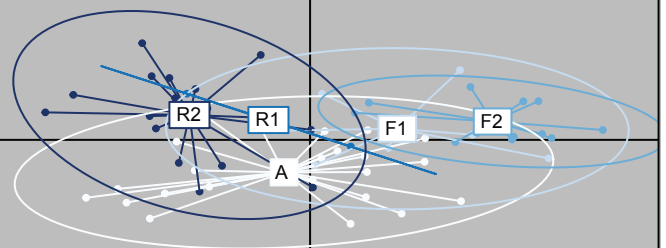

females

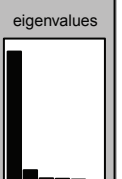

Supplement: Supplementary material 9 — Unconstrained ordination of wing band areas [file zookeys-540-489-s009.pdf]

# DAPC wing band areas

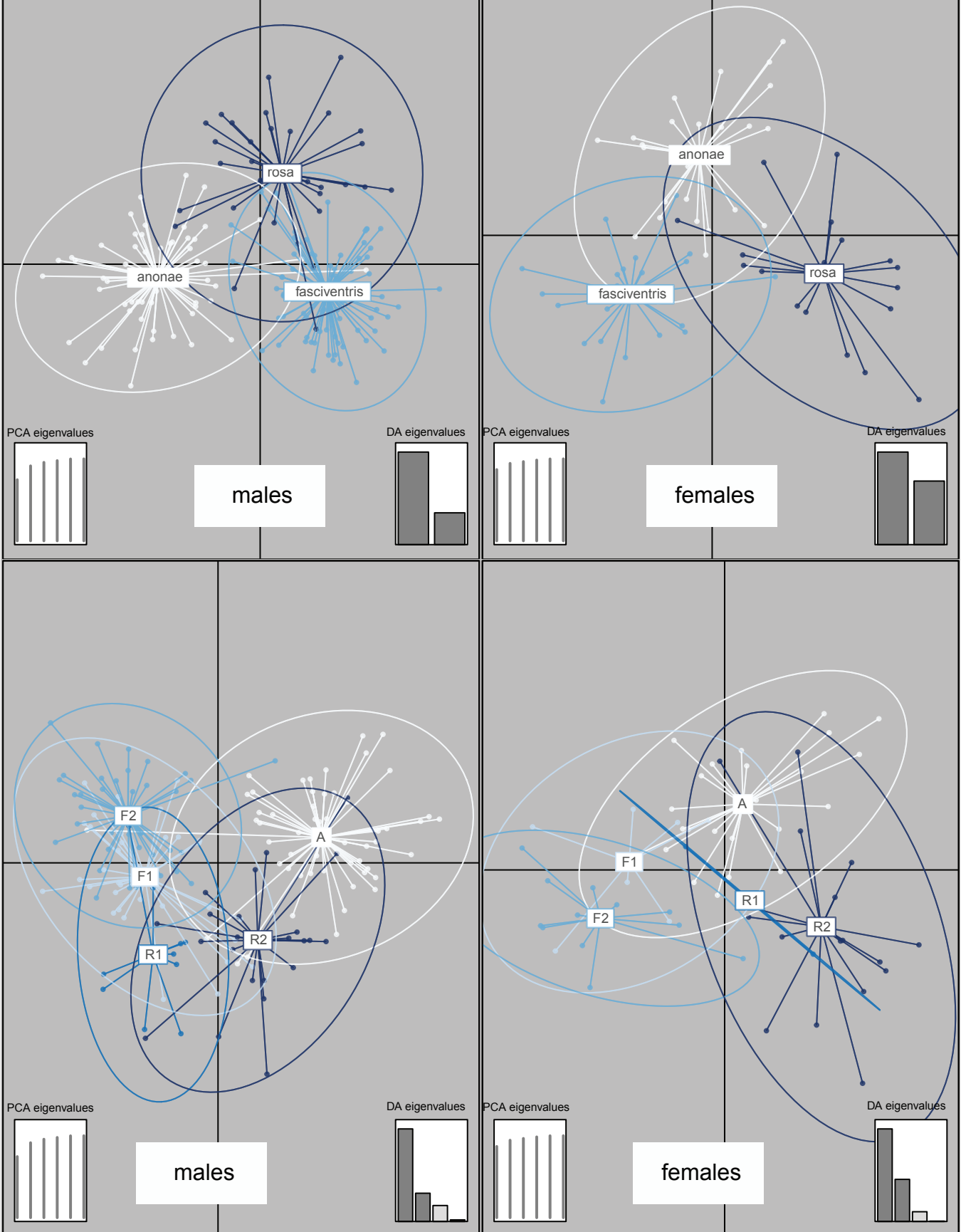

Supplement: Supplementary material 11 — Constrained ordination of wing band areas [file zookeys-540-489-s011.pdf]

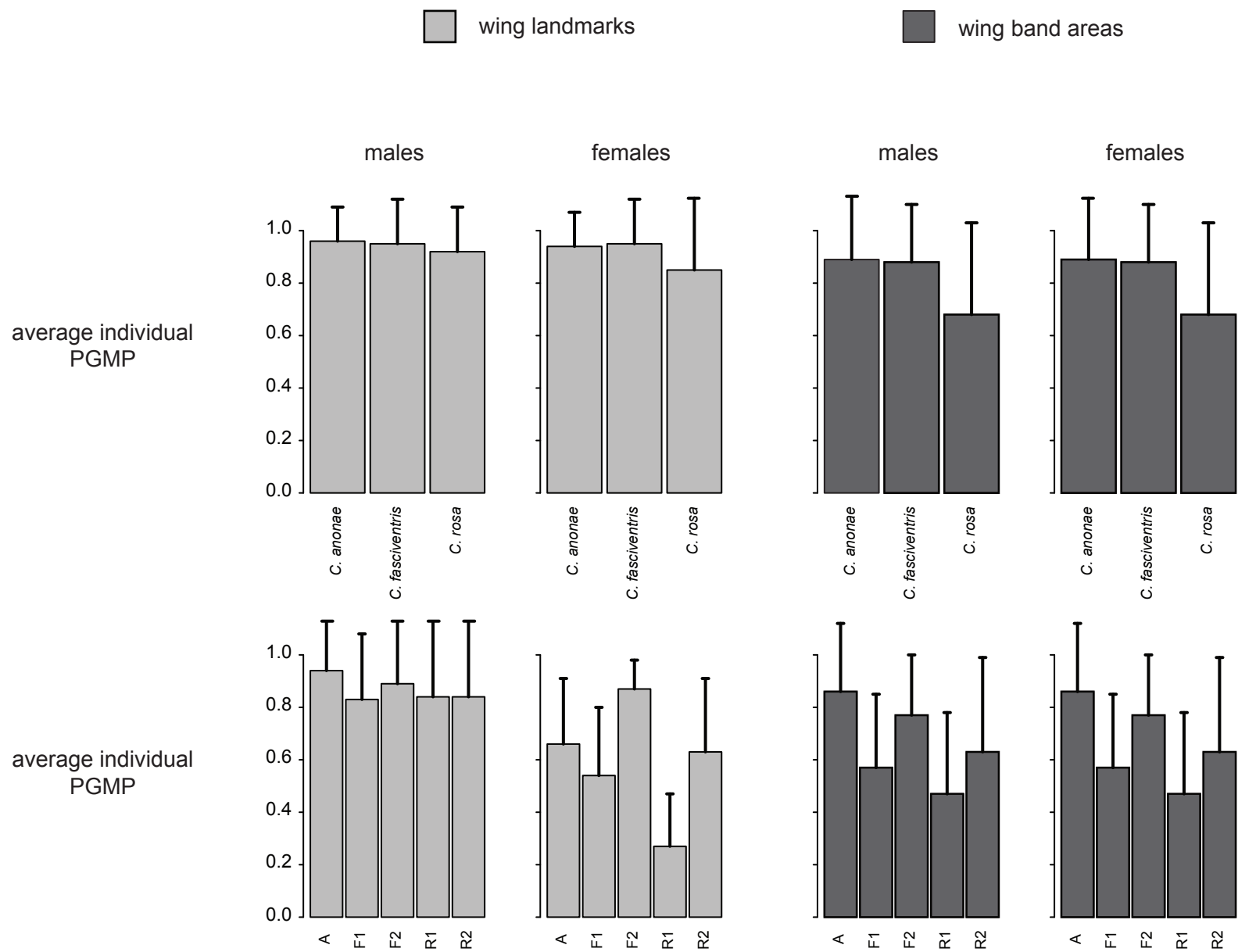

Supplement: Supplementary material 12 — Average individual assignments [file zookeys-540-489-s012.pdf]

## wing landmarks

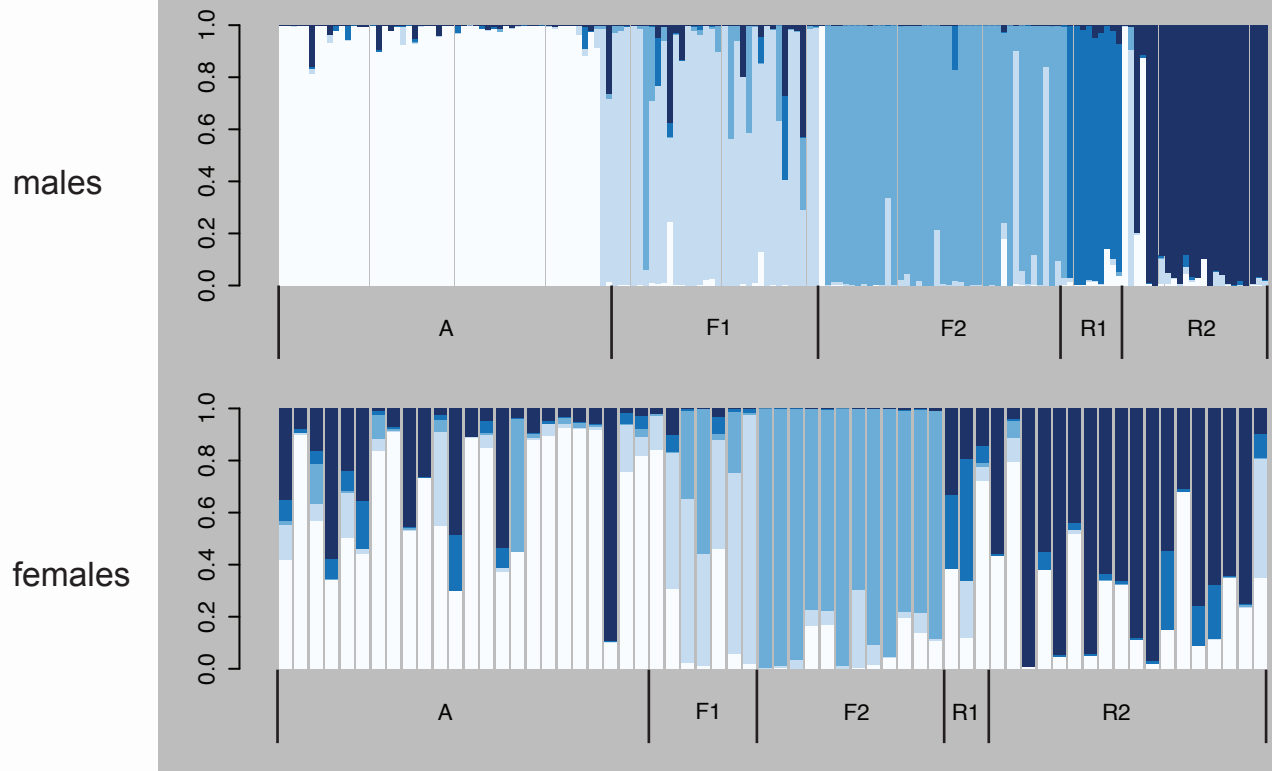

## wing band areas

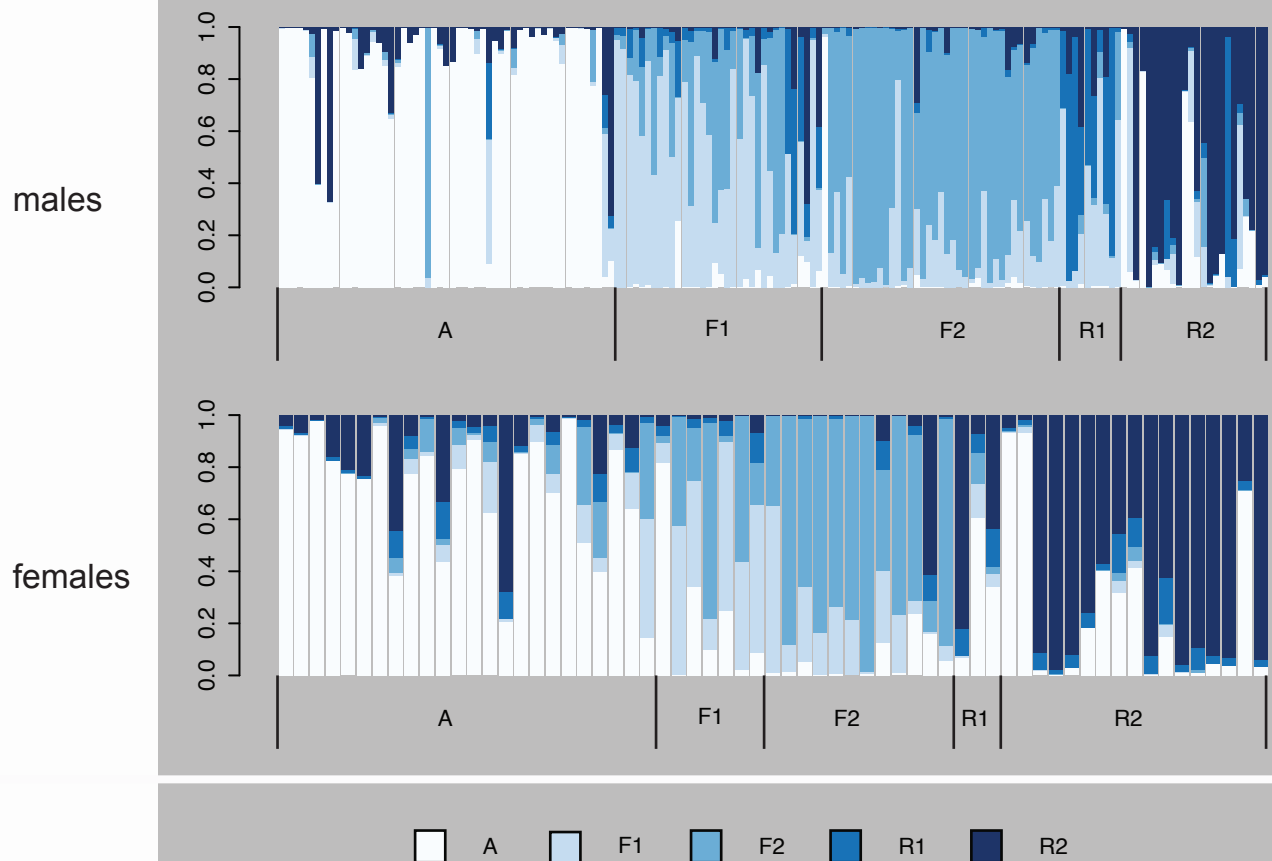

Supplement: Supplementary material 13 — Individual assignments to genotypic clusters A, F1, F2, R1, R2 [file zookeys-540-489-s013.pdf]
