# Supplementary material for: Wing morphometrics as a possible tool for the diagnosis of the Ceratitis fasciventris, C. anonae, C. rosa complex (Diptera, Tephritidae)
Source: Zookeys. 2015 Nov 26;(540):489–506. doi: 10.3897/zookeys.540.9724 (PMC4714084; doi:10.3897/zookeys.540.9724)
Supplement: Supplementary material 3 — Wing landmarks and wing band areas [file zookeys-540-489-s003.docx]

**wing landmarks**

1. Basal junction of veins of cell bm, inner side of cell bm
2. Anterio-anal corner of cell c
3. Anterio-costal corner of cell c
4. Costal junction of vein sc-r and vein Sc, inner side of cell sc
5. Junction of vein R1 and costal vein, inner side of cell sc
6. Termination of vein R2+3, inner side of cell r1
7. Termination of vein R4+5, inner side of cell r2+3
8. Termination of vein M, inner side of cell r4+5
9. Junction of vein CuA1 and dm-cu, inner side of cell dm
10. Junction of vein M and dm-cu, inner side of cell dm
11. Junction of vein M and r-m cross-vein, inner side of cell br
12. Junction of vein R4+5 and r-m cross-vein, inner side of cell br
13. Junction of veins R2+3 and R4+5, inner side of cell r2+3
14. Junction of vein M and dm-bm cross-vein, inner side of cell dm
15. Junction of vein CuA1 and dm-bm cross vein, inner side of cell dm
16. Junction of CuA1 and CuA2, inner side of cell bm
17. Junction of veins A1 and CuA2, inner side of cell cup

*Abbreviations for cells: bm= basal medial; c= costal; sc= subcostal; r1, r2, r3, r4, r5= radial; dm= discal medial; br= basal radial; cup= posterior cubicle. Abbreviations for veins and crossveins: Sc= subcostal; R1= anterior branch of radius; R2, R3, R4, R5= posterior (sectoral) branches of radius; CuA1, CuA2= anterior branches of cubitus; M= media; A1= branch of anal veins; dm-cu= discal medial-cubital; dm-bm= discal medial-basal medial; r-m= radial-medial. Wing landmarks 1, 5-12, 14-17 from Schutze et al., 2011*

**wing band areas**

1. Middle black spot within cell c

2. Black surface within cell sc

3. Anterior wingband within cell dm

4. Costal top of middle wingband within cell r4+5

5. Middle wingband within cell dm

6. Posterior wingband within cell r4+5

*Abbreviations for cells: c= costal; sc= subcostal; r4, r5= radial; dm= discal medial; br= basal radial*
