# Supplementary material for: Wing morphometrics as a possible tool for the diagnosis of the Ceratitis fasciventris, C. anonae, C. rosa complex (Diptera, Tephritidae)
Source: Zookeys. 2015 Nov 26;(540):489–506. doi: 10.3897/zookeys.540.9724 (PMC4714084; doi:10.3897/zookeys.540.9724)
Supplement: Supplementary material 8 — Unconstrained ordination of wing landmarks [file zookeys-540-489-s008.pdf]

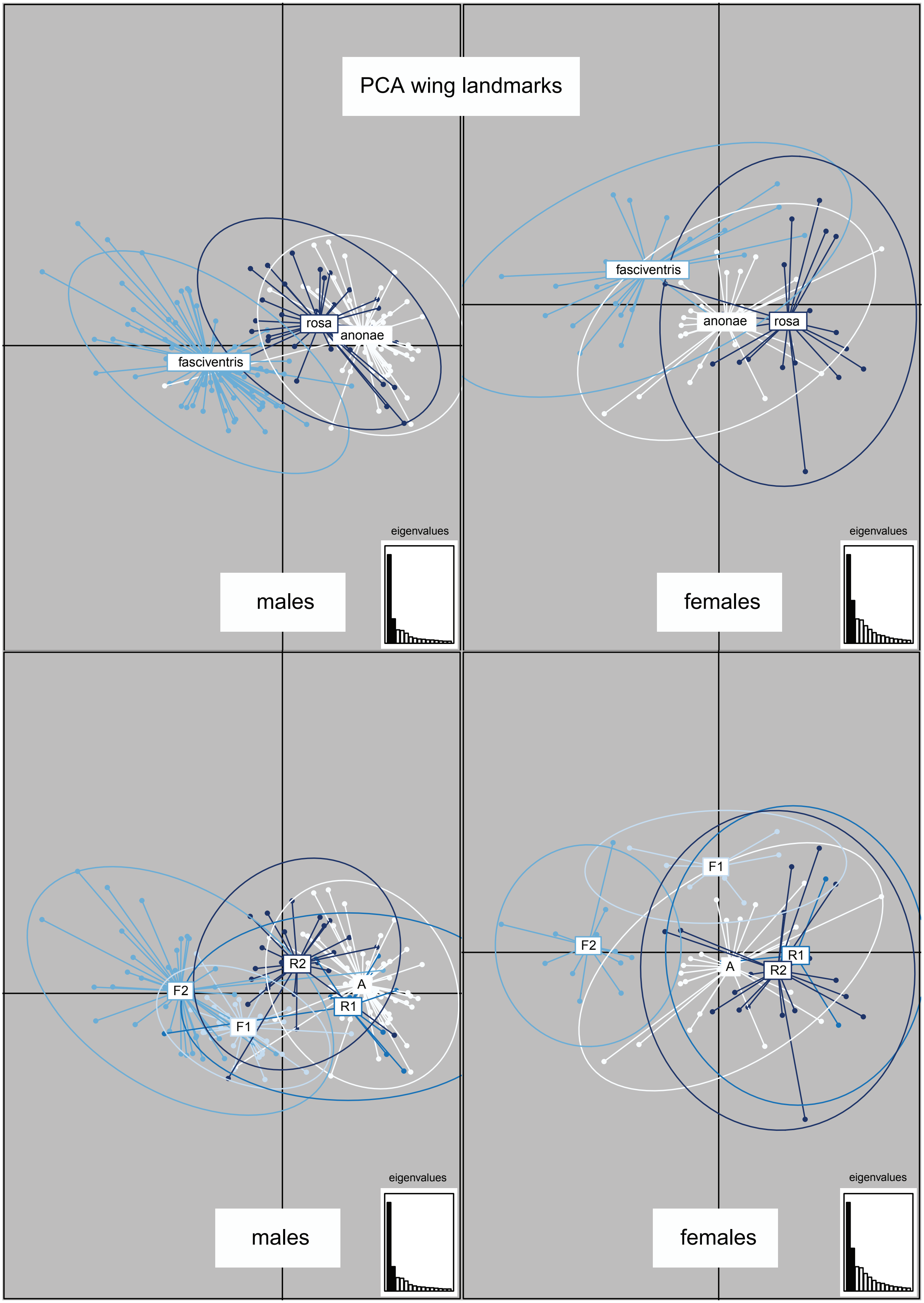

PCA wing landmarks
fasciventris
rosa
anonae
eigenvalues
males
fasciventris
anonae
rosa
eigenvalues
females
F2
R2
A
R1
F1
eigenvalues
males
F1
F2
A
R1
R2
eigenvalues
females
