# Supplementary material for: Wing morphometrics as a possible tool for the diagnosis of the Ceratitis fasciventris, C. anonae, C. rosa complex (Diptera, Tephritidae)
Source: Zookeys. 2015 Nov 26;(540):489–506. doi: 10.3897/zookeys.540.9724 (PMC4714084; doi:10.3897/zookeys.540.9724)
Supplement: Supplementary material 10 — Constrained ordination of wing landmarks [file zookeys-540-489-s010.pdf]

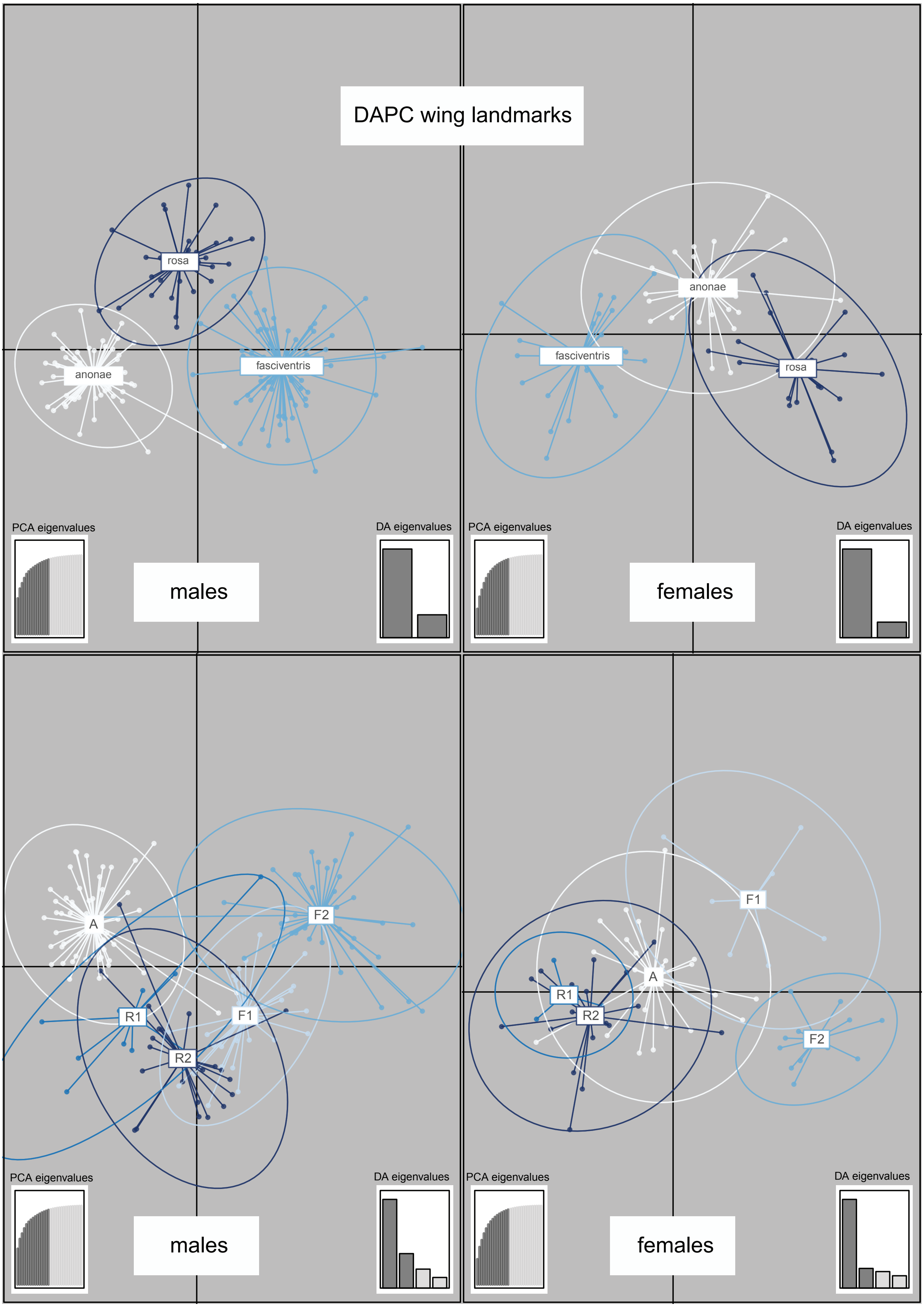
DAPC wing landmarks
rosa
anonae
fasciventris
PCA eigenvalues
DA eigenvalues
males
anonae
fasciventris
rosa
PCA eigenvalues
DA eigenvalues
females
A
F2
R1
F1
R2
PCA eigenvalues
DA eigenvalues
males
F1
A
R1
R2
F2
PCA eigenvalues
DA eigenvalues
females
