# Supplementary material for: Wing morphometrics as a possible tool for the diagnosis of the Ceratitis fasciventris, C. anonae, C. rosa complex (Diptera, Tephritidae)
Source: Zookeys. 2015 Nov 26;(540):489–506. doi: 10.3897/zookeys.540.9724 (PMC4714084; doi:10.3897/zookeys.540.9724)
Supplement: Supplementary material 14 — Morphometric differences across morphospecies (wing band areas) [file zookeys-540-489-s014.docx]

SF 13: Differences among morphospecies (wing band areas). PERMANOVA and *a posteriori* comparisons (t statistic) testing differences in multivariate patterns of wing band areas among morphospecies (*C. anonae*, *C. fasciventris*, *C. rosa*). d.f.: degrees of freedom; MS: mean square estimates; F: pseudo-F. Probability of Monte Carlo simulations: n.s.: not significant a P<0.05; ***: P<0.001, **: P<0.01; *: P<0.05 (after False Discovery Rate Correction for repeated *a posteriori* comparisons).

males females

d.f. MS F d.f. MS F

Morphospecies 2 0.0653 25.69 *** 2 0.0870 24.95 ***

Residual 90 0.0025 51 0.0035

Euclidean distances, untransformed data

Pair-wise *a posteriori* comparisons:

**Males**: *C. anonae* *C. fasciventris*

*C. fasciventris* ***

*C. rosa* *** ***

**Females**: *C. anonae* *C. fasciventris*

*C. fasciventris ****

*C. rosa ** ****
