# Supplementary material for: Wing morphometrics as a possible tool for the diagnosis of the Ceratitis fasciventris, C. anonae, C. rosa complex (Diptera, Tephritidae)
Source: Zookeys. 2015 Nov 26;(540):489–506. doi: 10.3897/zookeys.540.9724 (PMC4714084; doi:10.3897/zookeys.540.9724)
Supplement: Supplementary material 15 — Morphometric differences across genotypic clusters (wing band areas) [file zookeys-540-489-s015.docx]

SF 14: Differences among morphospecies (wing band areas). PERMANOVA and *a posteriori* comparisons (t statistic) testing differences in multivariate patterns of wing band areas among morphospecies (*C. anonae*, *C. fasciventris*, *C. rosa*). d.f.: degrees of freedom; MS: mean square estimates; F: pseudo-F. Probability of Monte Carlo simulations: n.s.: not significant a P<0.05; ***: P<0.001, **: P<0.01; *: P<0.05 (after False Discovery Rate Correction for repeated *a posteriori* comparisons).

males females

d.f. MS F d.f. MS F

Genotypic cluster 4 0.0271 13.67 *** 4 0.0253 12.32 **

Residual 45 0.0020 10 0.0021

Euclidean distances, untransformed data

Pair-wise *a posteriori* comparisons:

**Males** A F1 F2 R1

F1 ***

F2 *** *

R1 *** * ***

R2 n.s. *** *** n.s.

**Females** A F1 F2 R1

F1 *

F2 *** n.s.

R1 * n.s. *

R2 n.s. * *** n.s.
